# Aneurysm of left ventricle at non-atherosclerosis lesion of coronary arteries

**DOI:** 10.1186/1749-8090-10-S1-A154

**Published:** 2015-12-16

**Authors:** Olena Gogayeva, Gennadiy Knishov, Anatolii Rudenko, Liudmyla Dzakhoieva, Sergiy Rudenko, Kostyantyn Rudenko

**Affiliations:** 1 Department of Surgical Treatment of Ischemic Heart Disease, GF "National Amosov's Institute of Cardiovascular Surgery NAMS of Ukraine", Kyiv, 03110, Ukraine

## Background/Introduction

Aneurysm of left ventricle (ALV) is formed in each 5th patient with acute myocardial infarction in the presence of complete occlusion of the coronary artery (CA) by atherosclerotic plaque. But we noticed the formation of ALV in the absence of atherosclerotic stenosis, for patients with myocardial "bridges" (MB). The essence of this anomaly is the presence of systolic compression of the tunneled segment of the artery, which in itself raises doubts about its clinical significance. Due to the attitude of the medical community toward the MB, as a result of its ambiguous nature, and given the favorable long term trend, the MB is regarded as a variant of the norm. At the same time, increasing reports of cases of sudden death and myocardial infarction associated with the presence of MB demonstrates the relevance of this anomaly.

## Aims/Objectives

To show possibility of formation of postinfarction aneurism of left ventricle (LV) in the absence of atherosclerotic plaques in CA.

## Method

12 patients in average age 35+/-5 years with transmural MI in anamnesis underwent standard examination (ECG, ECHO and angiography) and surgical treatment.

## Results

All patients had ECG-signs of aneurysm of antero-septal and apical area of LV, which was confirmed by ECHO study, where we notice reduction of ejection fraction less 45% (from 35 till 45%). On the coronary angiography we found myocardial "bridge" (MB) over middle portion of LAD with systolic compression from 30% to 100% and aneurism of the apex of LV. We performed CABG with resection of an aneurism of LV with thrombectomy (in 7 cases) on-pump with good remote results after procedure.

## Discussion/Conclusion

Transient systolic compression of the LAD by MB can lead to myocardial infarction with the formation of ALV even in the absence of atherosclerotic lesions of CA.

